# Overexpression of the *Arabidopsis thaliana* signalling peptide TAXIMIN1 affects lateral organ development

**DOI:** 10.1093/jxb/erv291

**Published:** 2015-06-12

**Authors:** Janine Colling, Takayuki Tohge, Rebecca De Clercq, Geraldine Brunoud, Teva Vernoux, Alisdair R. Fernie, Nokwanda P. Makunga, Alain Goossens, Laurens Pauwels

**Affiliations:** ^1^Department of Plant Systems Biology, Flanders Institute for Biotechnology, (VIB), Technologiepark 927, B-9052 Gent, Belgium; ^2^Department of Plant Biotechnology and Bioinformatics, Ghent University, Technologiepark 927, B-9052 Gent, Belgium; ^3^Institute for Plant Biotechnology, Department of Genetics, Stellenbosch University, Stellenbosch, 7602, South Africa; ^4^Max-Planck-Institute of Molecular Plant Physiology, Am Mühlenberg 1, 14476, Potsdam-Golm, Germany; ^5^Laboratoire de Reproduction et Développement des Plantes, CNRS, INRA, ENS Lyon, Lyon, France; ^6^Department of Botany and Zoology, Stellenbosch University, Stellenbosch, 7602, South Africa

**Keywords:** Boundary genes, cysteine-rich peptide, fruit development, lateral organ fusion, paraclade junction.

## Abstract

Lateral organ boundary formation is highly regulated by transcription factors and hormones such as auxins and brassinosteroids. However, in contrast to many other developmental processes in plants, no role for signalling peptides in the regulation of this process has been reported yet. The first characterization of the secreted cysteine-rich TAXIMIN (TAX) signalling peptides in *Arabidopsis* is presented here. *TAX1* overexpression resulted in minor alterations in the primary shoot and root metabolome, abnormal fruit morphology, and fusion of the base of cauline leaves to stems forming a decurrent leaf attachment. The phenotypes at the paraclade junction match *TAX1* promoter activity in this region and are similar to loss of LATERAL ORGAN FUSION (LOF) transcription factor function. Nevertheless, *TAX1* expression was unchanged in *lof1lof2* paraclade junctions and, conversely, *LOF* gene expression was unchanged in *TAX1* overexpressing plants, suggesting TAX1 may act independently. This study identifies TAX1 as the first plant signalling peptide influencing lateral organ separation and implicates the existence of a peptide signal cascade regulating this process in *Arabidopsis*.

## Highlight

Overexpression of a new secreted cysteine-rich signalling peptide in *Arabidopsis* results in developmental defects, including lateral organ fusion. This finding suggests that organ boundary formation involves small peptide signalling.

## Introduction

Development of multicellular organisms requires tight spatiotemporal control of cell differentiation. In plants, this is established by gradients of morphogens, such as hormones or noncoding RNAs, and polypeptides, such as secreted peptides or mobile transcription factors (Vernoux *et al.*, 2010; Van Norman *et al.*, 2011; Skopelitis *et al.*, 2012; Long *et al.*, 2015). Two distinct classes of secreted peptides can be distinguished in plants: small post-translationally modified peptides such as the CLAVATA3/ENDOSPERM SURROUNDING REGION (CLE) family, and cysteine-rich peptides (CRPs), exemplified by the EPIDERMAL PATTERNING FACTOR (EPF) family (Murphy *et al.*, 2012). Although over 1000 secreted peptides have been predicted based on the *Arabidopsis* genome, only a handful have been characterized so far (Czyzewicz *et al.*, 2013).

CRPs are characterized by an N-terminal secretion signal and six or eight cysteines in the mature peptide, which are responsible for the internal disulfide bond formation required to establish the tertiary structure of the secreted molecule (Torii, 2012). Although other roles of secreted CRPs can be envisioned, it is assumed that most CRPs are involved in cell-to-cell signalling and are recognized by a receptor on a target cell membrane (Czyzewicz *et al.*, 2013). The best-studied ligand-receptor pathway for CRPs is the recognition of members of the EPF family by the membrane-localized ERECTA family of leucine-rich repeat receptor-like kinases. The ERECTA kinase can perceive multiple EPF family members that control distinct developmental pathways. In the epidermis, it perceives EPF1/EPF2 to restrict stomatal density (Hara *et al.*, 2007; Hara *et al.*, 2009), whereas in the phloem, it binds EPFL4/EPFL5/EPFL6 to determine inflorescence stem and pedicel length (Uchida *et al.*, 2012).

Other *Arabidopsis* CRPs that have been characterized include the AtLURE1 pollen tube attractant (Takeuchi and Higashiyama, 2012) and the S-locus Cys-Rich/S-locus Protein 11 self-incompatibility ligand (Indriolo *et al.*, 2012). Recently, a new CRP, TAXIMIN (TAX), was described from the yew tree, *Taxus baccata* (Onrubia *et al.*, 2014). The *TbTAX* gene was identified to be co-regulated with paclitaxel biosynthesis genes in elicited cell cultures, and codes for a secreted CRP that could modulate specialized metabolism in both *T. baccata* and *Nicotiana tabacum* (tobacco) (Onrubia *et al.*, 2014). However, the exact function of TbTAX, as well of its homologues in other plants such as *Arabidopsis*, remains unknown.

During plant growth, the shoot apical meristem (SAM) gives rise to the primary stem and all aboveground (lateral) organs. In the central zone of the SAM, slowly dividing stem cells give rise to daughter cells that first make up the peripheral zone, then the organ primordia, and finally differentiate into lateral organs, such as leaves, stems, and flowers (Gaillochet *et al.*, 2015). The meristem-to-organ boundary is a group of cells of distinct identity formed between the meristem and organ primordia and is characterized by specific gene expression profiles and morphology (Aida and Tasaka, 2006; Rast and Simon, 2008). Two roles can be attributed to meristem-to-organ boundaries: (i) the initial separation of the emerging organ of the meristem; and (ii) the production of new tissues later during development, such as of axillary meristems and carpel marginal meristems (Aida and Tasaka, 2006; Khan *et al.*, 2014).

Several proteins, mostly transcriptional regulators, have been reported to regulate boundary formation (Žádníková and Simon, 2014), with the CUP-SHAPED COTYLEDON (CUC) NAC transcription factors playing an important role. *CUC* genes are expressed at the boundary regions around organ primordia and are partially redundant in ensuring maintenance of the boundary (Heisler *et al.*, 2005; Hibara *et al.*, 2006). Loss of *CUC* gene function results in organ fusion, while gain-of-function mutants show an increased size of the boundary domain (Laufs *et al.*, 2004). Loss-of-function mutants of other genes specifically expressed in boundaries (boundary genes) are also often characterized by organ fusion defects (Žádníková and Simon, 2014). These include the transcription factors LATERAL ORGAN FUSION1 (LOF1) and LOF2 (Lee *et al.*, 2009), JAGGED LATERAL ORGANS (Borghi *et al.*, 2007), and BLADE ON PETIOLE1 and 2 (Ha *et al.*, 2007; Khan *et al.*, 2012).

In participation with these transcriptional regulators, polar auxin transport carriers also play a role in boundary establishment (Žádníková and Simon, 2014). Auxin gradients originating from the meristem and organ primordia intersect at the boundary, forming a local auxin minimum. ABCB19 is an ATP-binding cassette transporter required for normal basipetal auxin transport from the meristem auxin maximum (Noh *et al.*, 2001). Loss of ABCB19 function increases auxin levels in both meristems and boundary regions, disturbs the auxin minimum, and results in the fusion of cauline leaves to the primary stem and in pedicel–stem fusions, ultimately accompanied by reduced expression of boundary genes (Zhao *et al.*, 2013).

So far, no signalling peptides have been described to play a role in organ separation. Overexpression of the signalling peptide TAX1 in *Arabidopsis* is here reported to result in fusion of the cauline leaves to stems with only minor effects on the primary metabolome of leaf and root tissue. Accordingly, *TAX1* promoter activity is higher at the base of the cauline leaf and axillary stem and in the apical meristem. Interestingly, the developmental defects caused by *TAX1* overexpression are expanded in the Landsberg *erecta* (L*er*) background. Finally, although the *TAX1* overexpression phenotype at the paraclade junction phenocopies that of *lof1lof2* mutants, these data suggest that TAX and LOF signalling pathways converge independently.

## Materials and methods

### Plant materials and growth conditions

Plants in this study were either the Columbia (Col-0) or L*er* ecotype. For *in vitro* growth, seeds were gas-sterilized, stratified, and germinated on full-strength Murashige and Skoog (MS) medium (Murashige and Skoog, 1962). Plants were cultivated in a growth room at 22°C with a 16-h light/8-h dark photoperiod (110 µEm^-2^s^-1^). For analysis of adult plants, 10-day-old *in vitro*-germinated seedlings were transferred to soil in a growth chamber at 20–22°C and a photoperiod of 16-h light/8-h darkness.

T-DNA insertion lines for *TAX1* (SALK_016616) and *TAX2* (SALK_113004C) were obtained from Nottingham Arabidopsis Stock Center (NASC; Alonso *et al.*, 2003). Seedlings were PCR-genotyped using a T-DNA- and gene-specific primer (Supplementary Table S1). Amplicons were sequenced to confirm the location of the T-DNA.

### DNA constructs

The open reading frames of *Arabidopsis TAX1* (*At2g31090*), *TAX2* (*At2g20562*), and *TAX1∆SP* (lacking the N-terminal signal) were amplified between *attB* sites from Col-0 cDNA using Phusion polymerase (New England Biolabs) and Gateway recombined in the Entry vector pDONR207 (Invitrogen) and then in the pFAST-G02 destination vector (Shimada *et al.*, 2010) for overexpression. The *TbTAX* open reading frame was amplified from *T. baccata* cDNA and fused to 6xHis by PCR and cloned into the Entry clone pDONR221 (Invitrogen) and then in the pK7WG2D (Karimi *et al.*, 2005) destination vector. Promoter sequences of *TAX1* (1575bp upstream of the ATG) and *TAX2* (2000bp upstream of the ATG) were PCR amplified between *attB* sites from Col-0 gDNA, Gateway recombined in pDONRP4P1R and then in pmK7S*NFm14GW as the destination vector for promoter activity analysis (Karimi *et al.*, 2007). For subcellular localization, the *Venus* sequence was fused to that of *TAX1* and *TAX1∆SP* by PCR amplification and cloned as an entry clone in pDONR207 and recombined to destination vector pFAST-R02 (Karimi *et al.*, 2005; Shimada *et al.*, 2010).

### Plant transformation

All constructs were transformed into *Agrobacterium tumefaciens* strain C58C1 (pMP90) for subsequent transformation of Col-0 plants by floral dip (Clough and Bent, 1998). The 35S::TAX1 construct was used to transform both Col-0 and L*er* plants. Transformants were selected on MS media based on *OLE1:GFP* expression in seeds. Homozygous plant lines with one T-DNA locus were selected and used in all assays. All primers used for cloning are listed in Supplementary Table S1 at *JXB* online.

### Gene expression analysis

RNA was isolated from plant material using the Plant RNeasy Kit (Qiagen, Germany) following the manufacturer’s instructions with the addition of a DNase treatment step. cDNA was synthesized from 1 µg RNA using the iScript reverse transcriptase kit (Bio-Rad). Quantitative real-time PCR (qRT-PCR) was performed on a LightCycler 480 (Roche Applied Science, USA) using Fast Start SYBR Green I fluorescent dye (Roche). At least three biological repeats and three technical repeats were used for each analysis. Expression data were normalized through two reference genes, *UBC* (At5g25760) and *PP2A* (At1g13320). For RT-PCR, cDNA was amplified with the Go-Taq PCR mix (Promega) using different amplification cycles, and loaded on an agarose gel containing SYBR Safe (Life Technologies). The cycle number showing the highest contrast without saturation was used. *ACTIN* (At3g18780) was used as the reference gene for RT-PCR experiments. Primers for expression analysis are listed in Supplementary Table S1 at *JXB* online. Paraclade junctions (1cm of nodal tissue that includes the primary stem and part of the axillary stem and cauline leaf) were collected from eight plants to form one replicate and immediately frozen in liquid nitrogen.

### GUS expression

Plant material was harvested in 90% (v/v) acetone and kept at 4°C for up to 1 week to remove chlorophyll. For the GUS staining, plants were first rinsed in NT buffer (100mM Tris, pH 7.0 and 50mM NaCl) and incubated in ferricyanide solution [1.94mM potassium ferricyanide (K_3_[Fe(CN)_6_]) prepared in NT buffer] for 30min at 37°C. Next, plants were transferred to a staining solution (2.47mM X-Gluc prepared with ferricyanide solution) and kept at 37°C for at least 8 hours. Plants were kept in 70% (v/v) ethanol prior to visualization under a Bino Leica stereomicroscope (Leica MZ16) equipped with a digital camera.

### Microscopy

**Fig. 1.**
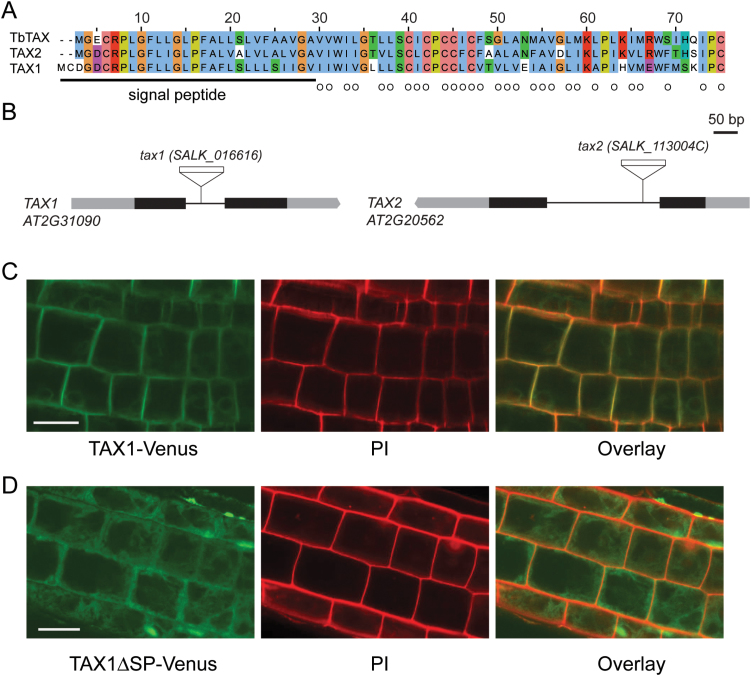
The TAXIMIN peptides in *Arabidopsis*. (A) Sequence alignment of TbTAX and *Arabidopsis* orthologues TAX1 and TAX2. The *in silico*-predicted TbTAX signal peptide (Onrubia *et al.*, 2014), located at the N-terminus, and hydrophobic amino acids are underlined and marked by circles, respectively. (B) Schematic diagram of the gene structure of *TAX1* and *TAX2* displaying the site of T-DNA insertion in the respective *tax* mutant lines. Black bars, grey bars, and black lines represent exons, UTR regions, and introns, respectively. (C, D) Subcellular localization of the TAX1 peptide with (C) and without (D) the N-terminal signal fused to Venus expressed in 5-day-old *Arabidopsis* root cells and visualized with a confocal microscope. Propidium iodide (PI) staining was used as a localization control. Scale bars are 20 µm.

Imaging of living SAMs of 5-week-old plants was performed using a LSM700 laser-scanning confocal microscope (Zeiss, Jena, Germany). TAX-Venus fusions were imaged in 5- or 10-day-old seedlings with an Olympus FV10 ASW confocal microscope. Images in Fig. 1 and Supplementary Fig. S1 at *JXB* online are from independent transformed lines. Before imaging, seedlings were briefly incubated in propidium iodide (3mg/L, Sigma) and subsequently washed and mounted in water. For scanning electron microscopy of gynoecia, flowers at stage 13 were collected from wild-type and *TAX1* overexpressing plants cultivated in the greenhouse. Sepals, petals, and stamen were removed to reveal the carpel, which was directly mounted on the steel stubs. Images were collected using a Hitachi TM-1000 table-top scanning electron microscope (Hitachi High-Technologies Corporation).

### Metabolite profiling

Metabolite profiling was performed exactly as described by Lisec *et al.* (2006), using the modifications for root tissue described in Joshi *et al.*, (2006). Metabolite identities were verified via comparison to spectral libraries of authentic standards housed in the Golm Metabolome Database (Kopka *et al.*, 2005). Metabolite information is provided following recent recommendation standards (Supplementary Table S2; Fernie *et al.*, 2011).

## Results

### The *TAX* genes encode putative signalling peptides

Recently, a novel putative signalling peptide termed TAXIMIN (TAX) was identified in the medicinal tree *T. baccata*. This peptide co-regulates with taxol biosynthesis genes and overexpression of *TbTAX* in *N. tabacum* hairy roots enhances production of alkaloids (Onrubia *et al.*, 2014). Analysis using the PLAZA comparative genomics workbench (Proost *et al.*, 2014) indicated that this peptide is highly conserved across the plant kingdom. Homologues with a remarkable sequence identity can already be found in the lower land plants *Selaginella moellendorffii* and *Physcomitrella patens* (Supplementary Fig. S1 at *JXB* online). In this study, the model species *Arabidopsis thaliana* was used to further characterize the function of the TAX signalling peptides, and two homologous sequences were discovered at the loci *At2g31090* and *At2g20562*, which were renamed *TAX1* and *TAX2*, respectively (Fig. 1A).

Both *Arabidopsis TAX* genes consist of two exons flanking one intron (Fig. 1B). *TbTAX* encodes a peptide of 73 amino acids (7.82kDa), whereas *TAX1* and *TAX2* encode 75 (8.15kDa) and 73 (7.84kDa) amino-acid peptides, respectively (Fig. 1A). The TAX2 peptide has the highest sequence similarity to TbTAX with 51 amino acids identical to its gymnosperm homologue (Fig. 1A).

TAXIMIN peptides have an *in silico*-predicted N-terminal secretion peptide (Fig. 1A, Onrubia *et al.*, 2014), generating equally sized mature peptides of 46 amino acids located at the C-terminus (Fig. 1A). The hydrophobicity of this mature peptide is striking, with up to 29 amino acid residues being hydrophobic. This hydrophobicity and equal mature peptide length is conserved in all TAXIMIN family members (Onrubia *et al.*, 2014). Currently, it is not known if and/or how the TAX peptides are post-translationally modified, but chemical synthesis of the TbTAX peptide was only possible when the prolines were hydroxylated (Onrubia *et al.*, 2014). The TAX peptides are cysteine-rich with six conserved cysteines and three conserved prolines (Fig. 1A), suggesting that this peptide belongs to the cysteine-rich family of peptides.

To validate the functionality of the predicted N-terminal signal peptide, fusions of TAX1 to the Venus fluorescent protein were constructed. TAX1-Venus with and without signal peptide were expressed with a 35S Cauliflower mosaic virus promoter in *Arabidopsis* seedlings and the localization of the fusion protein was determined by confocal microscopy in root cells. Similar to earlier observations of TbTAX subcellular localization (Onrubia *et al.*, 2014), TAX1-Venus was targeted to the plant cell membrane (Fig. 1C and Supplementary Fig. S1B at *JXB* online) and this was dependent on the presence of the N-terminal signal peptide (Fig. 1D and Supplementary Fig. S1C at *JXB* online).

It can be concluded that, like TbTAX, TAX1 is a putative signal peptide that is likely secreted through the canonical secretion pathway.

### *TAX1* overexpression causes developmental phenotypes

First, full-length *TAX1, TAX2*, and *TbTAX* were constitutively expressed under control of the 35S promoter in *Arabidopsis* ecotype Col-0. For each construct, several independent lines were selected for the presence of a single T-DNA locus and showing clear overexpression in seedlings.

**Fig. 2.**
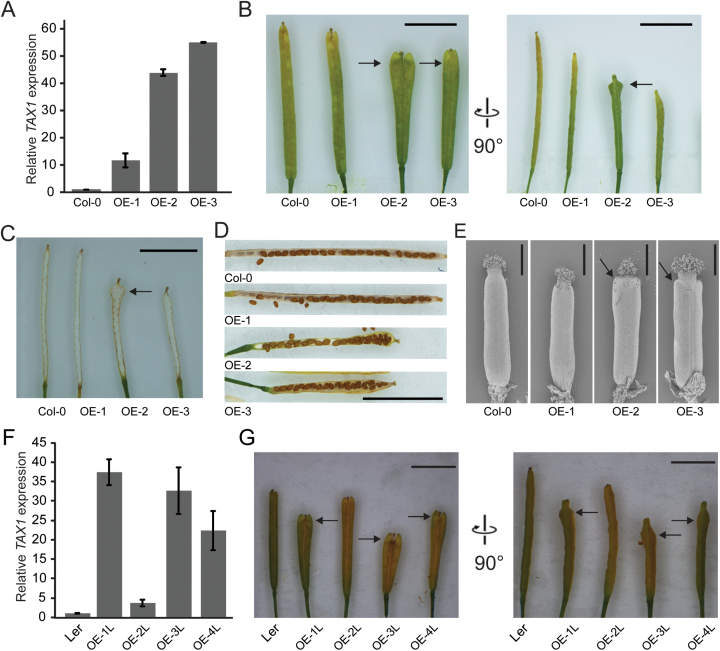
*TAX1* overexpression in Col-0 and L*er* backgrounds results in an alteration of fruit morphology. (A) Relative expression of *TAX1* in 19-day-old seedlings compared to the Col-0 wild type for three independent 35S::TAX1 lines. Expression values were normalized to those of the wild type (Col-0), set to 1. Values represent the average of three biological replicates ±SE. (B) Medial and lateral view of mature siliques of *TAX1* overexpression (OE) lines. (C) Lateral view with seeds removed. Arrow indicates protrusion of the replum at the tip of the silique in *TAX1* overexpressing lines. (D) Lateral silique view with one valve removed. (E) Scanning electron microscopy images of early stage gynoecia of *TAX1* OE lines. Arrow indicates protrusion. The scale bar is 400 µm. (F) Relative expression of *TAX1* in 19-day-old seedlings compared to the L*er* wild type for four independent 35S::TAX1 lines. Expression values were normalized to those of the wild type (L*er*). Values represent the average of four biological replicates ±SE. (G) Medial and lateral view of mature siliques of *TAX1* OE lines in the same order as in (F). Scale bars in B-D and G are 5mm.

**Fig. 3.**
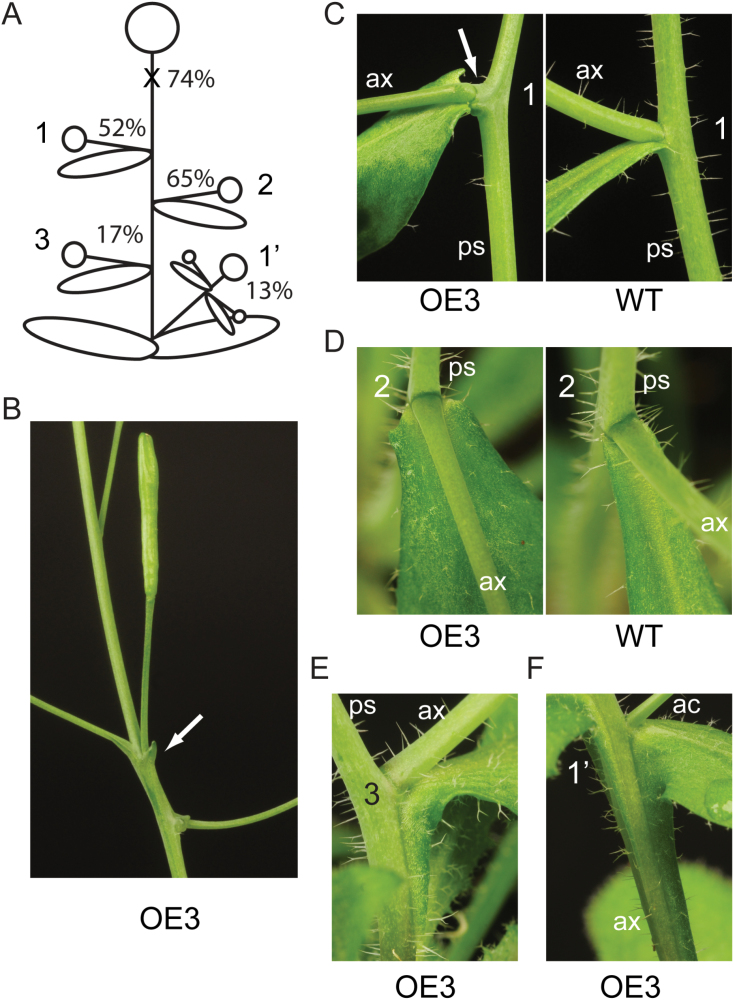
Constitutive *TAX1* overexpression results in lateral organ fusion with reduced penetrance. (A) Schematic overview of the location and frequency of phenotypes observed in line OE-3. (B) Undifferentiated outgrowths (indicated by arrow) at the inflorescence. (C) Side view of the protrusion (indicated by the arrow) of the primary stem at the first node, accompanied with fusion of the broader cauline leaf base to the axillary stem which deflects downwards. (D) Top view of node 2 with a broader cauline leaf base. (E) Fusion of the cauline leaf to both the axillary stem and the primary stem at node 3. (F) Fusion of cauline leaves to the axillary stem that originates from the rosette and to the accessory shoot when there is no third node. Abbreviations: ax, axillary stem; ac, accessory shoot; ps, primary stem.

The highest expressing *TAX1* overexpression lines OE-2 and OE-3 showed reduced growth of the seedlings on basal MS plates (Fig. 2A, Supplementary Fig. S2A at *JXB* online). After transfer to the greenhouse, these lines were delayed in development (Supplementary Fig. S2B, C at *JXB* online). Importantly though, *TAX1* overexpressing lines OE-2 and OE-3 showed developmental defects at paraclade junctions and altered fruit morphology (Figs 2 and 3).

Fruits of wild-type *Arabidopsis* Col-0 were narrow, cylindrical, and elongated (Fig. 2B). The siliques of the highest *TAX1* overexpressing lines OE-2 and OE-3 were shorter and wider at the tip, due to an outgrowth of both the valves (Fig. 2B) and the replum (Fig. 2C). The ovules inside the silique had a normal organization at the base of the fruit, but they were disordered at the wider tip (Fig. 2D). This was also associated with preferential opening of mature siliques at the site of seed crowding (Supplementary Fig. S2D at *JXB* online). The number of carpels in the 35S::TAX1 siliques was unaffected. The valve outgrowths were visible at early stages of gynoecium development (Fig. 2E) and could be followed over the course of fruit development (Supplementary Fig. S3 at *JXB* online).

The 35S::TAX1 transgene was also overexpressed in the L*er* background. Four lines were generated expressing *TAX1* at different levels (Fig. 2F) and similar fruit phenotypes were observed in these lines compared to the Col-0 background. Again, the severity correlated with *TAX1* expression levels (Fig. 2F, G). The silique phenotype observed in lines OE-2 and OE-3 in Col-0 and in OE-1L, OE-3L, and OE-4L in L*er* had complete penetrance: all siliques in all plants over several generations displayed this phenotype.

### *TAX1* constitutive expression results in lateral organ fusion

Besides the changes in fruit morphology, different types of lateral organ fusion were present in *TAX1* overexpressing lines at the paraclade junctions between the primary stem, axillary shoot, and cauline leaf (Fig. 3A). These phenotypes had reduced penetrance, and occurred in most plants of line OE-3 (Fig. 3A) and sporadically in OE-2 and L*er* (Supplementary Fig. S4 at *JXB* online). Of 23 OE-3 plants, 17 showed pedicel–stem fusions with outgrowths subtending some fruits, possibly corresponding to bract-like structures (Fig. 3B). At the first formed node on the main stem, 12 plants showed a protrusion of the main stem with the cauline leaf fused to a down- or side-wards deflected axillary shoot (Fig. 3C). At the second node, the cauline leaf had a broader leaf base and also deflected downwards (Fig. 3D). Finally, the most frequently occurring defect was a fusion of the cauline leaf to a stem together with the leaf extending down the insertion point along the stem, forming a decurrent leaf attachment. For the primary stem this was observed in 4 out of 23 plants (Fig. 3E). Interestingly, when no third node was visible, fusion occurred in 18 cases between a secondary shoot originating from the rosette, a tertiary axillary shoot, and the subtending cauline leaf (Fig. 3F). All these defects were associated with downward bending of the stems early during outgrowth of the axillary stem (Supplementary Fig. S2B at *JXB* online). The axillary stem bent upwards again later during development, probably owing to phototropism (Supplementary Fig. S2C at *JXB* online). No fusion of pedicels to stems was observed. In the L*er* background, additional phenotypes besides those seen in Col-0 were occasionally observed, such as bending of the axillary stem at tertiary branch points (Supplementary Fig. S4E at *JXB* online) and twisting of the primary stem (Supplementary Fig. S4F at *JXB* online).

### Lateral organ phenotypes are specific for *TAX1* overexpression

First, fruits and lateral organs of *Arabidopsis* lines overexpressing *TAX2* or *TbTAX* were examined (Supplementary Fig. S5A–E at *JXB* online). Although high transgene expression was detected, the lines displayed a wild-type phenotype. These results suggest that specifically TAX1 is capable of triggering organ fusion defects. *Arabidopsis* lines overexpressing *TAX2* did not display any visible phenotype in root or leaf growth.

Next, whether the signal peptide of TAX1 is essential for its activity was examined. A construct lacking the N-terminal signal, 35S::TAX1∆SP, was constitutively expressed, but failed to show any of the phenotypes observed for the full-length peptide (Supplementary Fig. S5F, G at *JXB* online). This result confirms that TAX1 represents a signalling peptide that is most likely secreted through the canonical pathway and that high peptide levels inside the cell do not result in observable phenotypes. Notably, wild-type phenotypes were observed in the TAX1-Venus lines (data not shown), suggesting that the fusion to Venus might interfere with TAX1 processing and/or function.

For both *TAX1* and *TAX2*, a T-DNA insertion line was isolated, *tax1* and *tax2* respectively, with the T-DNAs located in the sole intron (Supplementary Fig. S6A at *JXB* online). A cross was made between *tax1* and *tax2* to exclude functional redundancy between the two genes. Expression analysis by qRT-PCR in paraclade junctions (Supplementary Fig. S6B, C at *JXB* online) and RT-PCR in seedlings (Supplementary Fig. S6D at *JXB* online) confirmed the absence or at least severely reduced expression of both *TAX* genes in *tax1tax2.* Notwithstanding, single nor double mutant plants did not display any mutant phenotype in lateral organs or fruits (Supplementary Fig. S6E, F at *JXB* online). Likewise, no visible mutant phenotypes in root or leaf growth in *tax1* and *tax2* mutant lines could be observed.

### *TAX1* and *TAX2* have distinct expression patterns

The tissue-specificity of the promoter activities of *TAX1* and *TAX2* was then investigated. Promoter fragments of the 1575bp upstream of the start codon for *TAX1* and 2000bp upstream of the start codon for *TAX2* were cloned and used to drive a nuclear-localized GUS–GFP fusion (Karimi *et al.*, 2007).

**Fig. 4.**
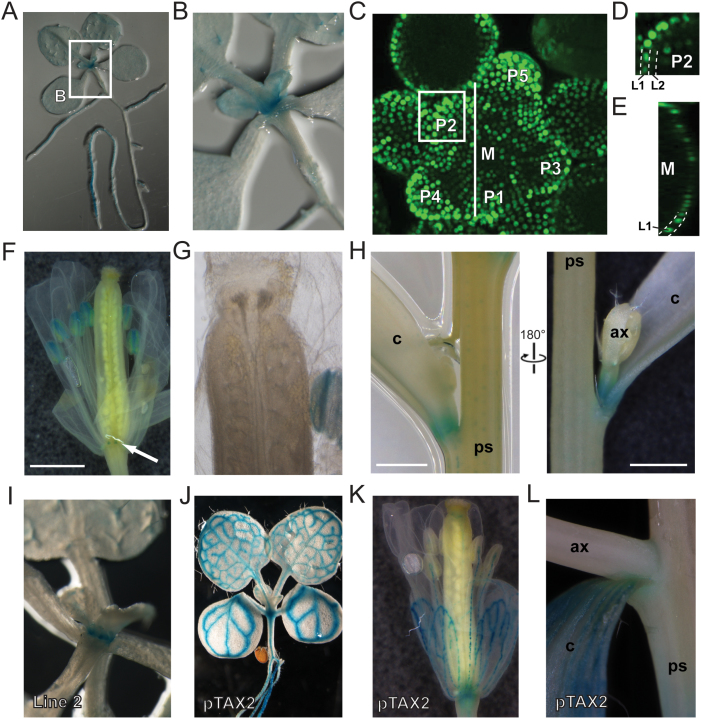
*TAX1* and *TAX2* have distinct promoter activities. Plants expressing a nuclear localized GUS-GFP fusion under control of the *TAX1* (A-I) and *TAX2* (J-L) promoter were used. (A, B) GUS activity in 10-day-old seedlings. (C-E) GFP signal at the shoot apical meristem visualized by confocal microscopy. (C) Stacked image. Primordia (P) are numbered from youngest to oldest. (D) Longitudinal optical section at P2. (E) Transverse optical section of the centre of the meristem (M). L1 and L2 layers are indicated. (F-H) GUS activity in the flower (F, G) and the paraclade junction (H) of 28-day-old mature plants. (I) GUS activity at the shoot apical meristem is also visible in a second *TAX1* reporter line that had lower *GUS* expression levels. (J-L) p*TAX2*::GUS activity in 10-day-old seedlings (J), the flower (K), and the paraclade junction (L) of 28-day-old mature plants. Abbreviations: ax, axillary stem; c, cauline leaf; ps, primary stem. All scale bars are 1mm.

In 10-day-old *in vitro*-germinated p*TAX1*::GUS:GFP seedlings, the *GUS* expression was detected mainly in the SAM region (Fig. 4A, B, I). Using GFP, *TAX1* promoter activity was detected in the entire inflorescence meristem of 5-week-old plants, but was stronger in the organ primordia (Fig. 4C-E). *TAX1* expression was mostly specific to the L1 layer in the centre of the meristem but was also detected in the L2 layer in organ primordia (Fig. 4C-E).

In mature plants cultivated for 28 days in the greenhouse, *TAX1* expression was observed in the anthers and in the nectaries in the floral tissue but no expression was visible in the gynoecium (Fig. 4F, G). The paraclade junctions between the primary stem and axillary stems showed *GUS* expression at the base of the cauline leaf and the emerging axillary shoot (Fig. 4H). The latter *TAX1* expression pattern supports a role of TAX1 in the lateral organ fusion phenotype observed following *TAX1* overexpression. Accordingly, no expression was observed in the pedicel–stem junctions, corresponding to the absence of fusion phenotypes there (Supplementary Fig. S7C at *JXB* online).

In contrast, in 10-day-old p*TAX2*::GUS:GFP seedlings, *GUS* was highly expressed in the vasculature in the cotyledons, first true leaves, and hypocotyl (Fig. 4J). In the floral tissue of mature plants, GUS was visible in the vasculature of the sepals, petals, and style (Fig. 4K). Also in paraclade junctions, GUS was detected mainly in the vasculature of the cauline leaf (Fig. 4L). For both *TAX1* and *TAX2*, GUS was observed in main and lateral roots in seedlings, mainly in vasculature, but was absent from the root tip (Supplementary Fig. S7 at *JXB* online). Expression in root hair cells was only observed for *TAX2* (Supplementary Fig. S7B, G at *JXB* online).

Overall, the difference in *TAX1* and *TAX2* expression patterns suggests that they play distinct roles in plant development, which is in agreement with the different effects caused by their overexpression.

### Expression of known boundary genes does not change in *TAX1* overexpression lines

The fusion of cauline leaves to the stem has been previously reported in *LOF* loss-of-function lines (Lee *et al.*, 2009) or to be associated with reduced *LOF* expression in *abcb19* (Zhao *et al.*, 2013). The expression of *LOF1* and *LOF2* in junctions of the *TAX1* overexpressing lines was therefore determined. The boundary gene *CUC3* was also included, because it has been reported to be downstream of the LOF transcription factors and because loss of CUC3 function causes fusion defects (Hibara *et al.*, 2006).

**Fig. 5.**
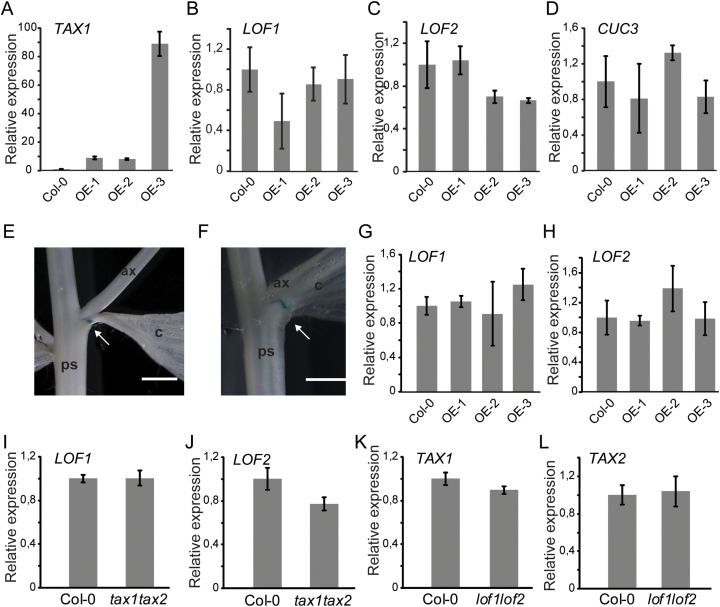
Expression of lateral organ boundary genes does not change in *TAX1* overexpressing lines. (A-D) Relative expression of *TAX1* and boundary genes in the paraclade junctions of 35S::TAX1 lines (TAX1 OE abbreviated as OE) cultivated in the greenhouse for 5 weeks. Expression values were normalized to those of the wild type (Col-0), set to 1. Values represent the average of three biological replicates ±SE. (E, F) GUS activity in paraclade junctions of 28-day-old mature p*LOF2*::GUS (E) and p*LOF2*::GUS x *TAX1* OE-3 (F). Arrows indicate the *LOF2* expression domain. ax, axillary stem; c, cauline leaf; ps, primary stem. Scale bar = 1mm. (G, H) *LOF1* (G) and *LOF2* (H) expression in 19-day-old seedlings. (I, J) *LOF1* (I) and *LOF2* (J) expression in paraclade junctions of 5-week-old *tax1tax2* plants. (K, L) *TAX1* (K) and *TAX2* (L) expression in paraclade junctions of 5-week-old *lof1lof2* plants.

First, overexpression of *TAX1* in the junctions was confirmed by qRT-PCR (Fig. 5A). Line OE-3 showed very high expression of *TAX1* at this site, corresponding to the severity of the fusion phenotypes observed in these lines associated with downward bending of the axillary stem (Fig. 3). However, expression of none of the tested boundary genes was significantly altered at this site (Fig. 5B-D). To confirm this finding, a p*LOF2*::GUS reporter line (Lee *et al.*, 2009) was crossed into the OE-3 background. Both the expression intensity and pattern of p*LOF2*-driven expression remained unaltered under *TAX1* overexpression (Fig. 5E, F), confirming the qRT-PCR results. Likewise, also earlier during development, *LOF* expression was unchanged in seedlings overexpressing *TAX1* (Fig. 5G, H). Finally, when expression of *LOF1* and *LOF2* was tested in paraclade junctions of the *tax1tax2* double mutant, no changes could be observed (Fig. 5I, J). Conversely, to assess whether *TAX1* or *TAX2* could act downstream of the LOF transcription factors, the paraclade junctions of *lof1lof2* mutant plants that show similar fusion defects (Lee *et al.*, 2009) were harvested, but, again, no significant effect on *TAX1* and *TAX2* expression could be detected (Fig. 5K, L).

These expression data suggest that the similar phenotypes observed for *TAX1* overexpression lines and *lof1lof2* are independent and probably result from converging signalling pathways.

### *TAX1* overexpression has only minor effects on the leaf and root metabolome

Having documented developmental phenotypes for the *TAX1* overexpressors, and given the link of the *T. baccata* homologue *TAXIMIN* with plant metabolism (Onrubia *et al.*, 2014), an established GC-MS protocol was used to assess whether *TAX1* overexpressing seedlings contained changes in the levels of primary metabolites in their leaves (Supplementary Table S3) and roots (Supplementary Table S4).

**Fig. 6.**
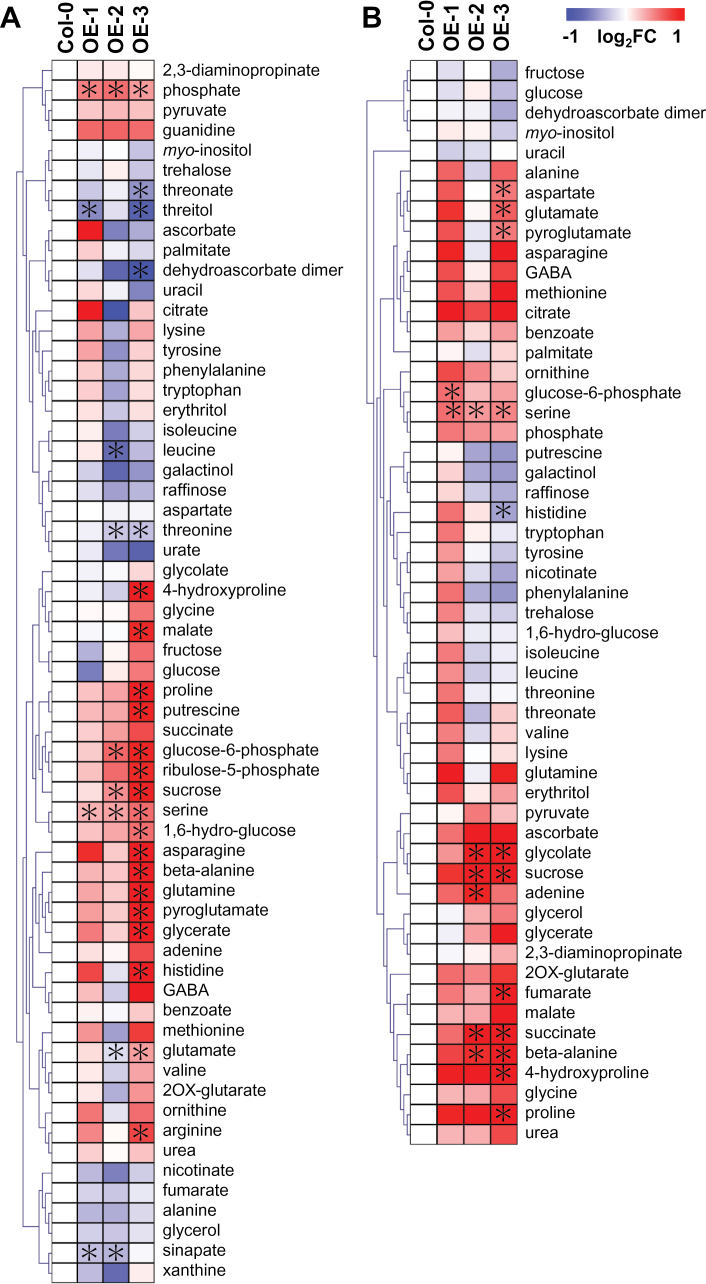
*TAX1* overexpression results in minor alterations of primary metabolites in leaf and root. Metabolic changes in *TAX1* overexpressing (OE) lines in (A) leaf and (B) root were visualized by heat-map. Analysis was performed with four independent biological replicates. Relative peak area was normalized by internal standard and fresh weight. Fold change against Col-0 is shown with a logarithmic scale. Fold change is visualized by colour codes, with red and blue indicating higher and lower, respectively. Hierarchical clustering by Pearson correlation was conducted using MeV software (http://www.tm4.org/mev.html). Asterisk represents statistical significance (*P* < 0.05).

The results of these analyses are presented in the heat-map of Fig. 6. Four, eight, and 22 of 61 measured metabolites were significantly different in the leaves of weak (OE-1), intermediate (OE-2), and strong (OE-3) *TAX1* overexpressing lines, respectively, with only phosphate and serine being altered (enhanced in both instances) in all three lines. That said, sucrose and glucose 6-phosphate were increased in both line OE-2 and line OE-3, whilst threonine was significantly decreased in both lines. Line OE-3 was additionally characterized by increased levels of histidine, putrescine, glucose, 4-hydroxyproline, ribulose 5-phosphate, asparagine, pyroglutamate, glutamine, glycerate, β-alanine, proline, malate, glutamate, and arginine. In contrast, this line displayed decreased levels of dehydroascorbate, threonate, and threitol. In roots, the changes were even less marked, with two, six, and 10 of 61 metabolites significantly different in the weak, intermediate, and strong overexpressing lines, respectively, and only serine being altered (again enhanced) in all three lines. That said, glycolate, succinate, sucrose, and β-alanine were increased in both line OE-2 and line OE-3, whilst hydoxyproline, pyroglutamate, glutamine, proline, aspartate, and fumarate were increased and histidine decreased only in line OE-3.

## Discussion

More than 1000 signalling peptides, most of them still uncharacterized, are encoded in the *Arabidopsis* genome (Czyzewicz *et al.*, 2013). A first characterization of a family of two *Arabidopsis* peptides, TAX1 and TAX2, the homologues of the TbTAX (Onrubia *et al.*, 2014), has been presented here. TbTAX was shown to localize to the plasma membrane through the secretory system and ectopically activate specialized metabolite pathways in yew and tobacco cells (Onrubia *et al.*, 2014).

### Regulatory neofunctionalization of the *Arabidopsis TAX* genes

Because of the relative sequence similarity between *Arabidopsis TAX1* and *TAX2*, possible functional redundancy was investigated. During evolution, a paralogue may have lost all functionality or gained a new function. Other paralogues share the ancestral function or remain redundant in *Arabidopsis* (De Smet and Van de Peer, 2012). The promoters of the *Arabidopsis TAX* genes have very distinct expression patterns, with *TAX2* being expressed mainly in the vasculature and *TAX1* rather at specific sites, such as anthers and nectaries in flowers, the paraclade junction on the primary stem, and the L1 layers of the SAM, implying at least regulatory neofunctionalization of the two *Arabidopsis TAX* genes. Furthermore, overexpression of *TAX1*, but not of *TAX2* or *TbTAX*, led to severe developmental phenotypes, suggesting functional specialization of the *Arabidopsis* TAX peptides, with roles in diverging target pathways. The pronounced effects caused by *TAX1* overexpression on the *Arabidopsis* developmental programmes that are reported here raise the possibility that the effect of TbTAX on metabolism might be indirect and caused by preceding developmental rewiring. Accordingly, the biosynthesis of paclitaxel in *Taxus* spp. is tissue-dependent (Vidensek *et al.*, 1990) and overexpression of *TbTAX* in tobacco hairy roots leads to morphological changes (Onrubia *et al.*, 2014).

### *TAX1* overexpression mimics loss of LOF function

Several developmental phenotypes are apparent in *TAX1* overexpressing lines at paraclade junctions: (i) cauline leaves are partially fused to stems; (ii) the cauline leaf base is widened and can form a decurrent strand extending down along the primary stem; and (iii) axillary stems bend downwards before growing upwards again, probably due to phototropism. The severity of these phenotypes was associated with the *TAX1* expression levels in the paraclade junctions. Similar phenotypes have been described for the *lof1-1* and *lof1-1lof2-1* mutants (Lee *et al.*, 2009). These lines are defective in the closely related MYB-domain transcription factors LOF1 and LOF2, which are expressed in *Arabidopsis* organ boundaries. In *lof1-1*, the cauline leaves are also fused to the axillary branch at the base, which bends down and completely lacks accessory shoots (Lee *et al.*, 2009). Additional phenotypes that are also presented in *TAX1* overexpressing lines, such as the decurrent leaf attachment, were only observed in the *lof1-1lof2-1* double mutant.

Nonetheless, no reduction in *LOF1* and *LOF2* expression levels in *TAX1* OE paraclade junctions or seedlings was observed. Therefore, down-regulation of *LOF1* and *LOF2* is not causal for the phenotype. Conversely, *TAX1* expression was not de-regulated in *lof1-1lof2-1* paraclade junctions, suggesting that high *TAX1* expression was not causal for fusion defects in this line. Another boundary gene that has been linked to fusion of cauline leaves to the primary stem is *CUC3* (Hibara *et al.*, 2006). *CUC3* expression was also down-regulated in *lof1-1* and *lof1-1lof2-1* paraclade junctions (Lee *et al.*, 2009) and *LOF1* expression was down-regulated in the *cuc3-105* mutant (Gendron *et al.*, 2012). The *cuc3-105* allele also enhanced paraclade fusions in the *lof1-1* background (Lee *et al.*, 2009). However, *CUC3* expression did not change in paraclade junctions of the *TAX1* overexpression lines. Fusion phenotypes are often observed in loss–of-function mutants for genes expressed in the boundary (Žádníková and Simon, 2014). However, promoter activity in the SAM shows that *TAX1* is not a boundary gene itself. The fact that the most well-known boundary genes affecting fusion of cauline leaves to neighbouring organs do not show a change in expression in *TAX1* overexpressing lines suggests that TAX1 might work in a converging, yet unknown, signalling pathway.

### *TAX1* overexpression also affects fruit development

*TAX1* overexpression also resulted in shorter siliques with protrusions at the tip of both valve tissue and the replum resulting in seed crowding. Basal parts of the fruit were however normal. In contrast to lateral organ separation, there is a known role for plant peptides in *Arabidopsis* fruit development. For instance, the CLE peptide family was discovered due to the club-shaped fruit of the *clv3* mutant, resulting from an enlarged flower meristem and extra floral whorls (Clark *et al.*, 1996). Overexpression of members of the *DEVIL* (*DVL*)*/ROTUNDIFOLIA4* family resulted in an alteration of silique morphology with different members causing different phenotypes, including protrusions at the tip in *DVL1* overexpressors (Wen *et al.*, 2004). Although the exact cellular and molecular bases of these phenotypes are currently not well understood, *DVL1* expression was associated with down-regulation of the valve identity regulator *AGAMOUS-LIKE8* (*AGL8*)*/FRUITFUL* (Wen *et al.*, 2004). Several organ-meristem boundary genes also influence fruit development. Gain-of-function lines of *CUC1* and *CUC2* prevent congenital fusion of carpels (Nikovics *et al.*, 2006; Sieber *et al.*, 2007; Larue *et al.*, 2009) and the *LOF1* gain-of-function line *constricted fruit 1* displays small misshapen fruits with increased replum size and enhanced expression of the valve margin identity markers *SHATTERPROOF1* (*SHP1/AGL1*) and *SHP2/AGL5* (Gomez *et al.*, 2011). The effect of *TAX1* overexpression on fruit development could therefore be linked to its influence on lateral organ separation.

### *TAX1* overexpression mildly affects the primary metabolome

Given the link between TbTAX and plant metabolism (Onrubia *et al.*, 2014), the primary metabolome of *TAX1* overexpressing seedlings was profiled. The changes observed in the primary metabolites were comparatively mild in the overexpressors with only two of the changes conserved across the genotype in leaves, namely phosphate and serine, and only one (again serine) in the roots. However, the extent of metabolic change was consistent with the degree of overexpression and the severity of the developmental phenotypes in the lines.

When assessed at a pathway level, the leaf data clearly suggest an elevated rate of photosynthesis on a per gram fresh weight basis, with increases in pentose- and hexose-phosphates as well as in sucrose. In addition, the intimately connected pathway of photorespiration appears to be up-regulated as indicated by the above-mentioned increases in serine and also in glycerate. The enhanced levels of phosphate would also be anticipated to facilitate the operation of photosynthesis, which can be phosphate-limited *in vivo*, suggesting that the increase in sucrose was not due to an inhibition of sucrose export driven by the lower sink strength, but rather indicative of an increased rate of sucrose synthesis. Of note, but only in the strongest line, was a general increase in the levels of the amino acids intimately associated with the TCA cycle. Such changes have previously been observed following increases in leaf sucrose (see for example Purdy *et al.*, 2013) and have been noted to invoke changes in the levels of some phytohormones, such as gibberellic acid (Araújo *et al.*, 2012). However, these metabolic changes were only seen in line OE-3 and not in line OE-2, which has a very similar, albeit less severe, developmental phenotype, and as such it is difficult to envisage them being causal of these phenotypes. Similar arguments preclude a strong case for a role of putrescine in the determination of the phenotype, despite considerable evidence being presented that this metabolite can exhibit bioactivity (Handa and Mattoo, 2010).

The root data presented fewer metabolic differences; however, two were highly notable. First, consistent with recent reports on the functionality of the enzymatic reactions of photorespiration in roots (Nunes-Nesi *et al.*, 2014), considerable changes were seen not only in serine but also in glycolate within this tissue. However, given that the exact function of these reactions in root tissue is currently not established, the significance of this observation remains unclear. Second, a much clearer up-regulation of the TCA cycle intermediates and closely associated metabolites was observed, albeit only significantly in the strongest overexpressing line. Previous work on tomato lines exhibiting reduced expression of any of the TCA cycle enzymes revealed that this resulted in decreased root growth, most likely as a compound result of decreases in cell wall biosynthesis and an alteration in the balance of phytohormone levels (van der Merwe *et al.*, 2009).

### Peptide signalling in lateral organ separation

Additional phenotypes were caused by *TAX1* overexpression in the L*er* background compared to Col-0, such as bending at a tertiary branch point and twisting of the stem. It has been reported that the L*er* ecotype influences lateral organ phenotypes. For example, the L*er* background possibly increases organ fusion between the cauline leaf and the axillary stem in *lob* mutants defective in the transcription factor LATERAL ORGAN BOUNDARIES (LOB) (Bell *et al.*, 2012). LOB negatively regulates brassinolide (BR) biosynthesis in organ boundaries by activating the expression of the *BAS1* gene encoding a BR-inactivating enzyme. Consequently, loss of LOB leads to hyperaccumulation of BR in the boundary (Bell *et al.*, 2012). Similarly, hyperactivation of BR signalling in the *bzr1-D* mutant or BR treatment also leads to fusion of the cauline leaf to the axillary stem and is associated with bending of the primary stem (Gendron *et al.*, 2012). The *bzr1-D* mutation constitutively activates the BZR1 transcription factor, capable of targeting the promoters of a plethora of genes, including *CUC3* (Gendron *et al.*, 2012; Guo *et al.*, 2013). Accordingly, not only *CUC3* expression, but also *LOF1* expression was reduced in *bzr1-D* paraclade junctions by BR treatment (Gendron *et al.*, 2012). Further work will be required to determine the relationship between *TAX1* overexpressing phenotypes and hormone signalling.

The lack of any obvious phenotype for a *tax1* loss-of-function mutant raises the possibility of functional redundancy or that overexpression of *TAX1* leads to ectopic receptor activation (Rowe and Bergmann, 2010; Torii, 2012). Notwithstanding, the presented work implicates the existence of a peptide signal cascade regulating lateral organ separation in *Arabidopsis*.

## Supplementary data

Supplementary data are available at *JXB* online.

Supplementary Fig. S1. Distribution of the TAXIMIN peptide family in the plant kingdom.

Supplementary Fig. S2. Phenotypes of *TAX1* overexpressing seedlings and flowering plants.

Supplementary Fig. S3. Fruit developmental series of Col-0 and *TAX1* overexpression lines.

Supplementary Fig. S4. Paraclade junction phenotypes in line OE-2 and L*er* background with reduced penetrance.

Supplementary Fig. S5. Effects of constitutive overexpression of *TAX2*, *TbTAX-His*, or *TAX1dSP* in *Arabidopsis thaliana*.

Supplementary Fig. S6. Generation and characterization of *tax* loss-of-function lines.

Supplementary Fig. S7. *TAX1* and *TAX2* expression.

Supplementary Table S1. Primers used in this study

Supplementary Table S2. Reporting metabolite data presented in this study.

Supplementary Table S3. Primary metabolite profiling of Col-0 and *TAX1* overexpressing lines in leaf.

Supplementary Table S4. Primary metabolite profiling of Col-0 and *TAX1* overexpressing lines in root.

Supplementary Data

## Abbreviations:

ABC transporter: ATP-binding cassette transporter
BR: brassinolide
CLE: CLAVATA3/ENDOSPERM SURROUNDING REGION
CRPs: cysteine-rich peptides
CUC: CUP-SHAPED COTYLEDON
EPF: EPIDERMAL PATTERNING FACTOR
LOB: LATERAL ORGAN BOUNDARIES
LOF: LATERAL ORGAN FUSION
qRT-PCR: quantitative real-time polymerase chain reaction
SAM: shoot apical meristem
TAX: TAXIMIN
WT: wild type.
