# Supplementary material for: Overexpression of the Arabidopsis thaliana signalling peptide TAXIMIN1 affects lateral organ development
Source: J Exp Bot. 2015 Jun 12;66(17):5337–49. doi: 10.1093/jxb/erv291 (PMC4526920; doi:10.1093/jxb/erv291)
Supplement: Supplementary Data [file supp_66_17_5337__index.html]

Overexpression of the Arabidopsis thaliana signalling peptide TAXIMIN1 affects lateral organ development — Supplementary Data 

# Overexpression of the *Arabidopsis thaliana* signalling peptide TAXIMIN1 affects lateral organ development

## Supplementary Data

Data files

- Supplementary Data - Supplementary Data
- Supplementary Data - Supplementary Data
- Supplementary Data - Supplementary Data
- Supplementary Data - Supplementary Data
