# Supplementary material for: Overexpression of the Arabidopsis thaliana signalling peptide TAXIMIN1 affects lateral organ development
Source: J Exp Bot. 2015 Jun 12;66(17):5337–49. doi: 10.1093/jxb/erv291 (PMC4526920; doi:10.1093/jxb/erv291)
Supplement: Supplementary Data [file supp_erv291_JEXPBOT_147926_Supplementary_Figures.pdf]

Supplementary data

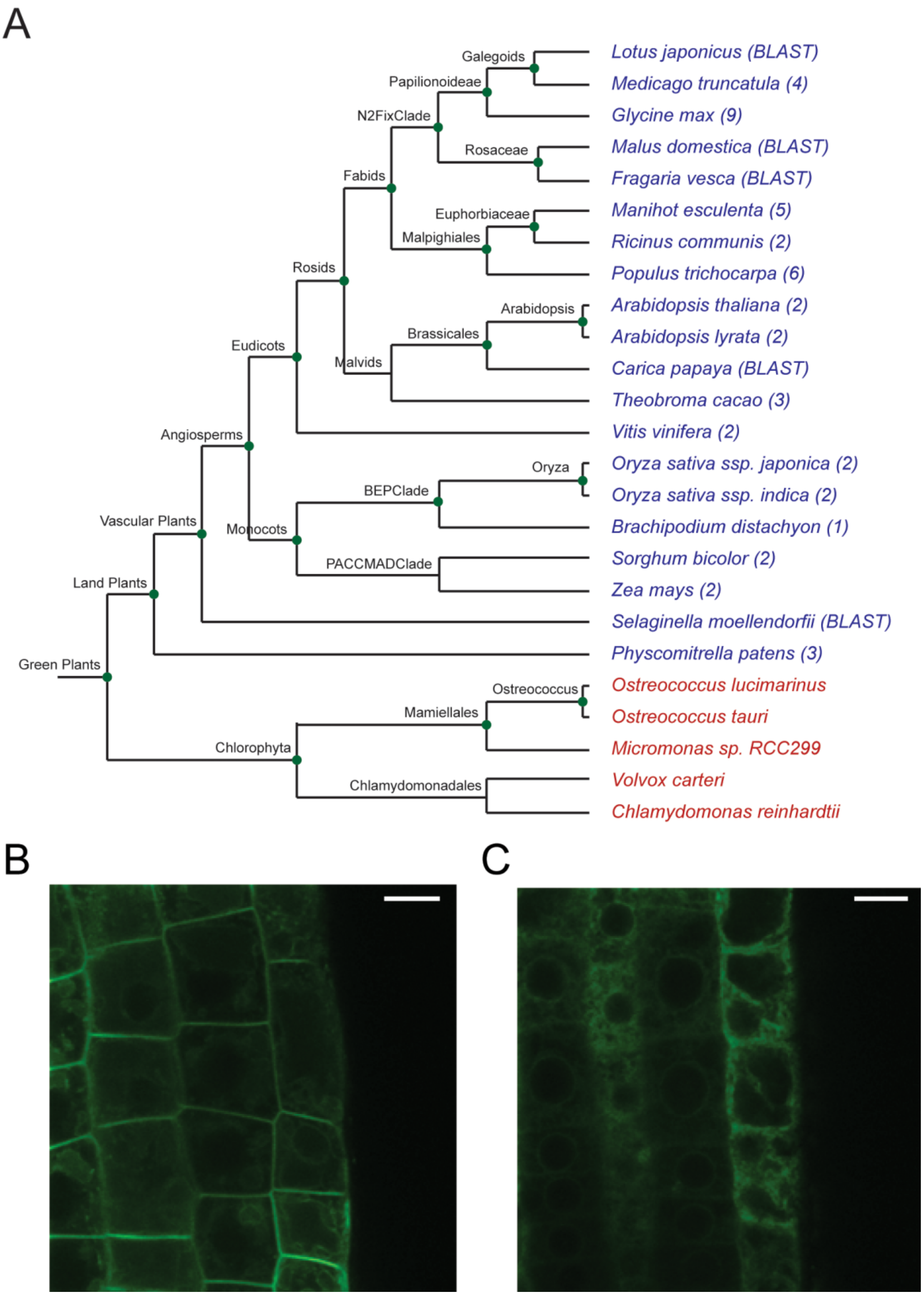

Supplementary Fig. S1. Distribution of the TAXIMIN peptide family in the plant kingdom. (A) Phylogenetic analysis Species indicated in blue and red indicate the presence and absence, respectively, of a homologue found in the species based on data from the PLAZA3.0 comparative genomics platform. The numbers between brackets indicate the number of homologues that were detected in each species in the PLAZA3.0 platform or by BLASTp. (B-C) Subcellular localization of the TAX1 peptide with (B) and without (C) the N-terminal signal fused to Venus expressed in 10-day-old Arabidopsis root cells and visualized with a confocal microscope. Scale bars are 10 µm.

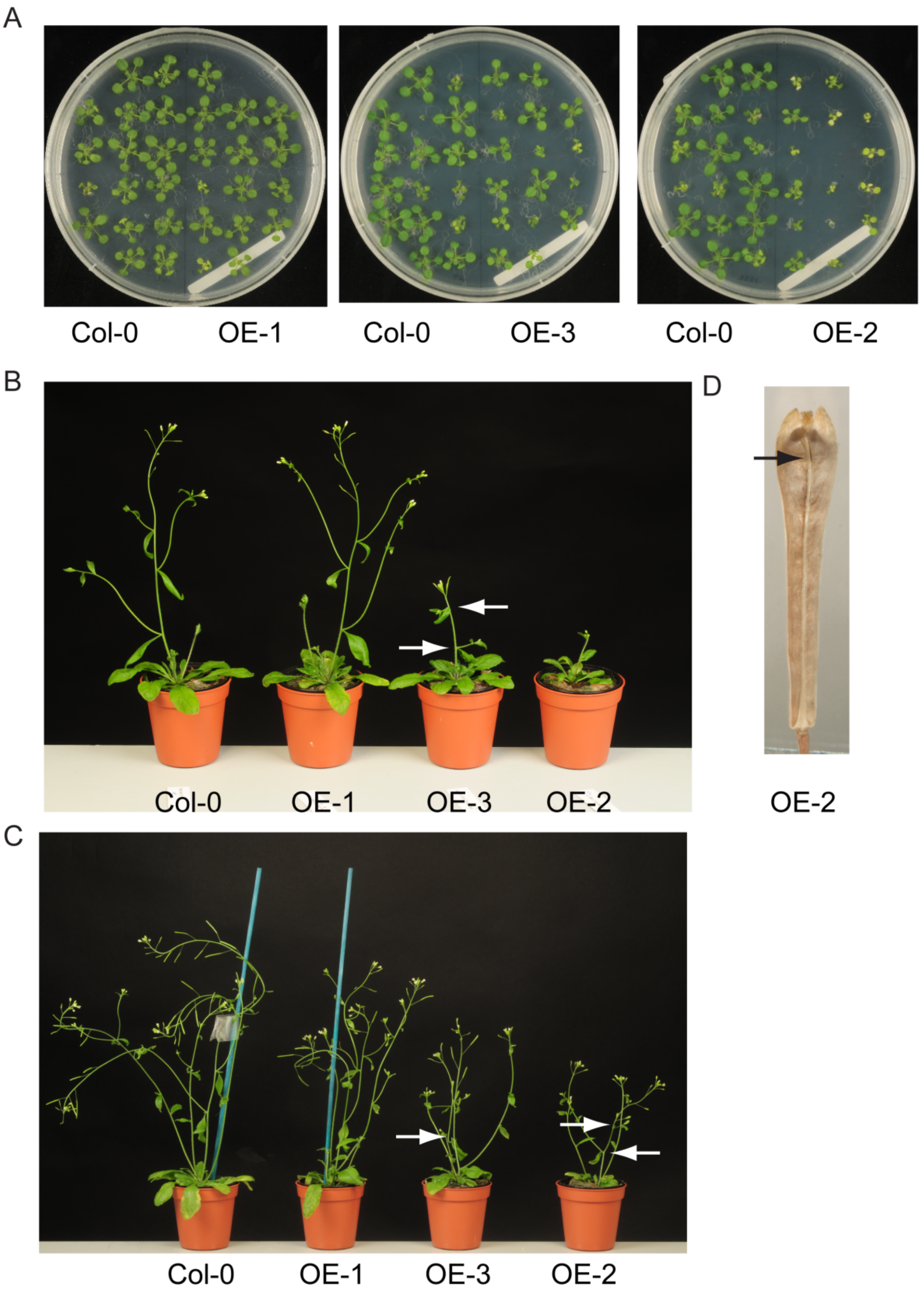

Supplementary Fig. S2. Phenotypes of *TAX1* overexpressing seedlings and flowering plants. (A) Seedlings (21-day-old) germinated on basal MS. Left and right sides of each plate are wild type Col-0 and the *TAX1* overexpressing lines, respectively. (B) Flowering plants (5-week-old) grown with 16-h light in a growth chamber. Arrows indicate the bending of the axillary shoots in OE-3 which proceeded to grow upwards after 5 days (C, arrow in OE-3) At this age, also phenotypes in OE-2 at the inflorescence (top arrow) and the first node (bottom arrow) are visible (C). (D) Siliques of line *TAX1* OE-2 opening in the middle due to seed crowding.

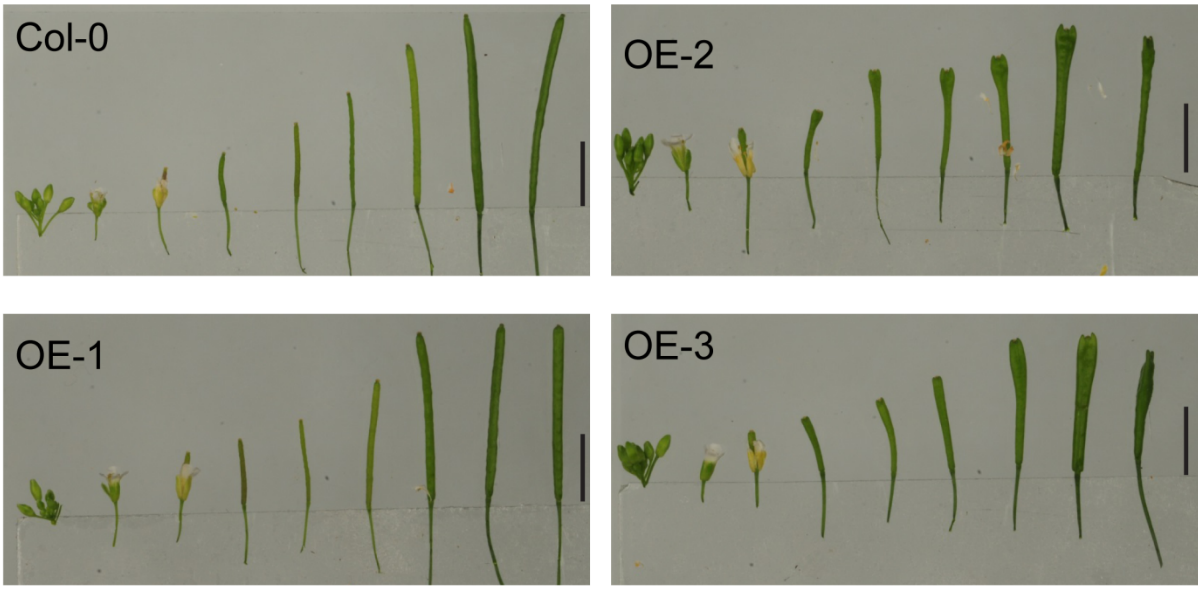

Supplementary Fig. S3. Fruit developmental series of Col-0 and *TAX1* overexpression lines. All fruits in a series are imaged in medial view, except for the last one, which is in lateral view. Scale bars: 5 mm.

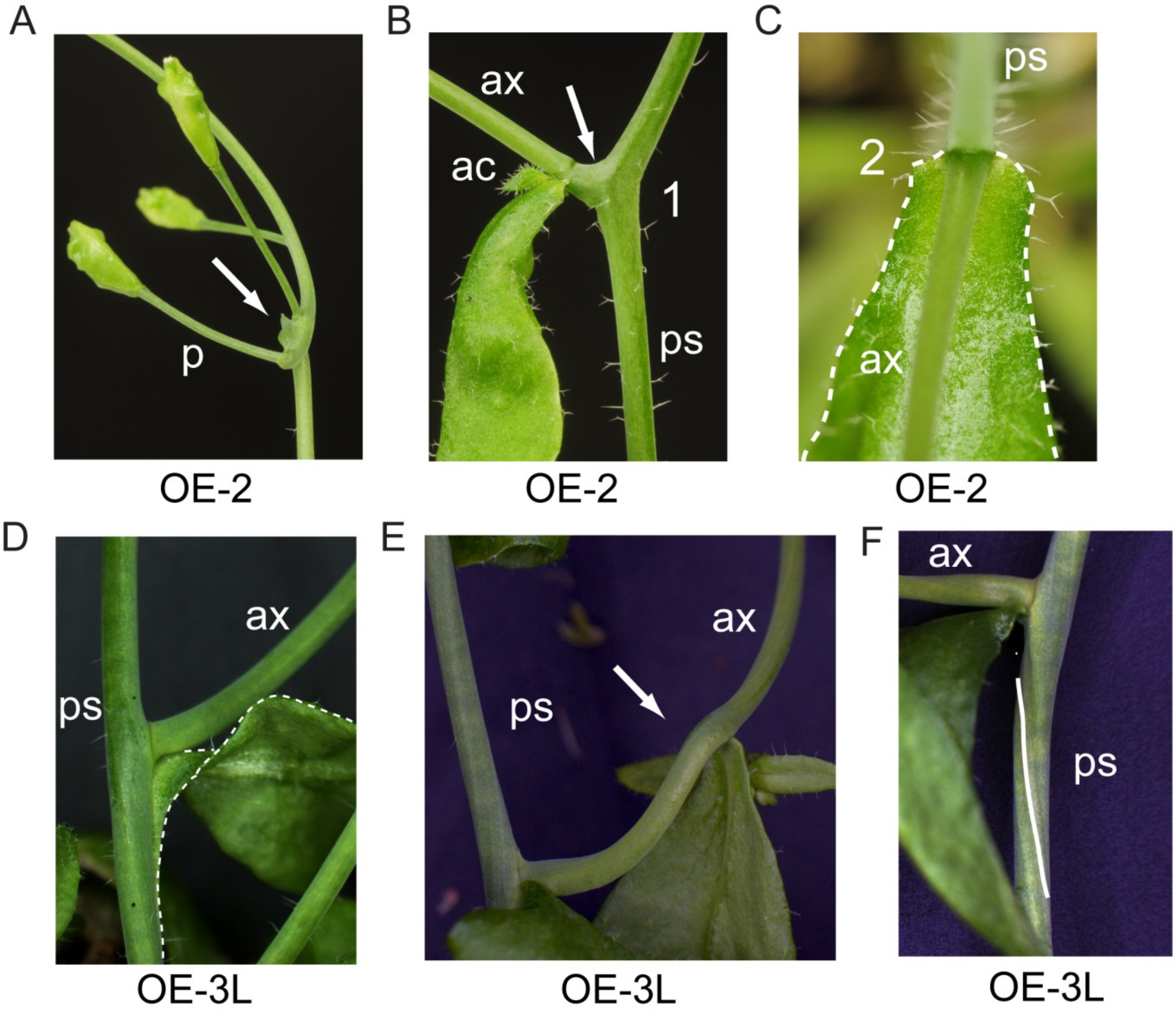

Supplementary Fig. S4. Paraclade junction phenotypes in line OE-2 and L*er* background with reduced penetrance. (A) Undifferentiated outgrowths (indicated by arrow) at the inflorescence. (B) Side view of the protrusion (indicated by arrow) of the primary stem at the first node. (C) Top view of node 2 with a broader cauline leaf base. (D) Fusion of the cauline leaf to both the axillary stem and the primary stem in L*er* background. Dashed lines indicate contours of the cauline leaf. (E) Additional bending of the axillary stem at tertiary branch points (arrow). (F) Twisting of the primary stem. The white line follows the stem. Abbreviations: ps, primary stem; ax, axillary stem; ac, accessory shoot; p, pedicel.

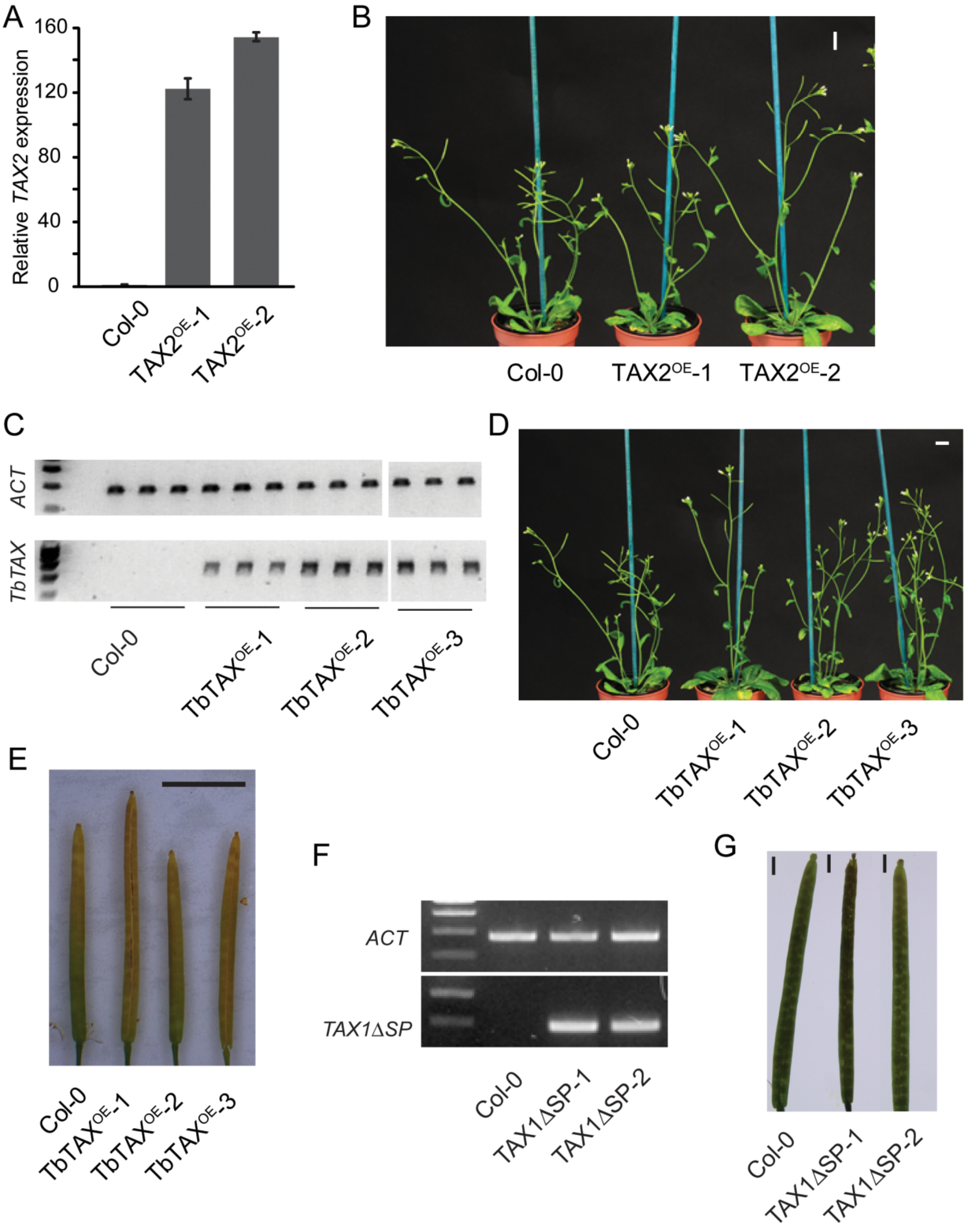

Supplementary Fig. S5. Effects of constitutive overexpression of *TAX2*, *TbTAX-His* or *AtTAX1dSP* in *Arabidopsis thaliana*. (A) Relative expression of *TAX2* determined by qRT-PCR. The y-axis indicates fold overexpression relative to the wild type Col-0 (set to 1). (B) General morphology of *TAX2* overexpressing plants. (C) Confirmation of *TbTAX-His* overexpression by RT-PCR. (D) General morphology of *TbTAX-His* overexpressing plants. (E) Silique morphology of *TbTAX-His* overexpressing plants. (F) Confirmation of *TAX1ΔSP* overexpression by RT-PCR. (G) Silique morphology of *TAX1ΔSP* overexpressing plants. Scale bars in (B and D) are 12 mm, in (E) it is 5 mm and in (G) it is 1 mm.

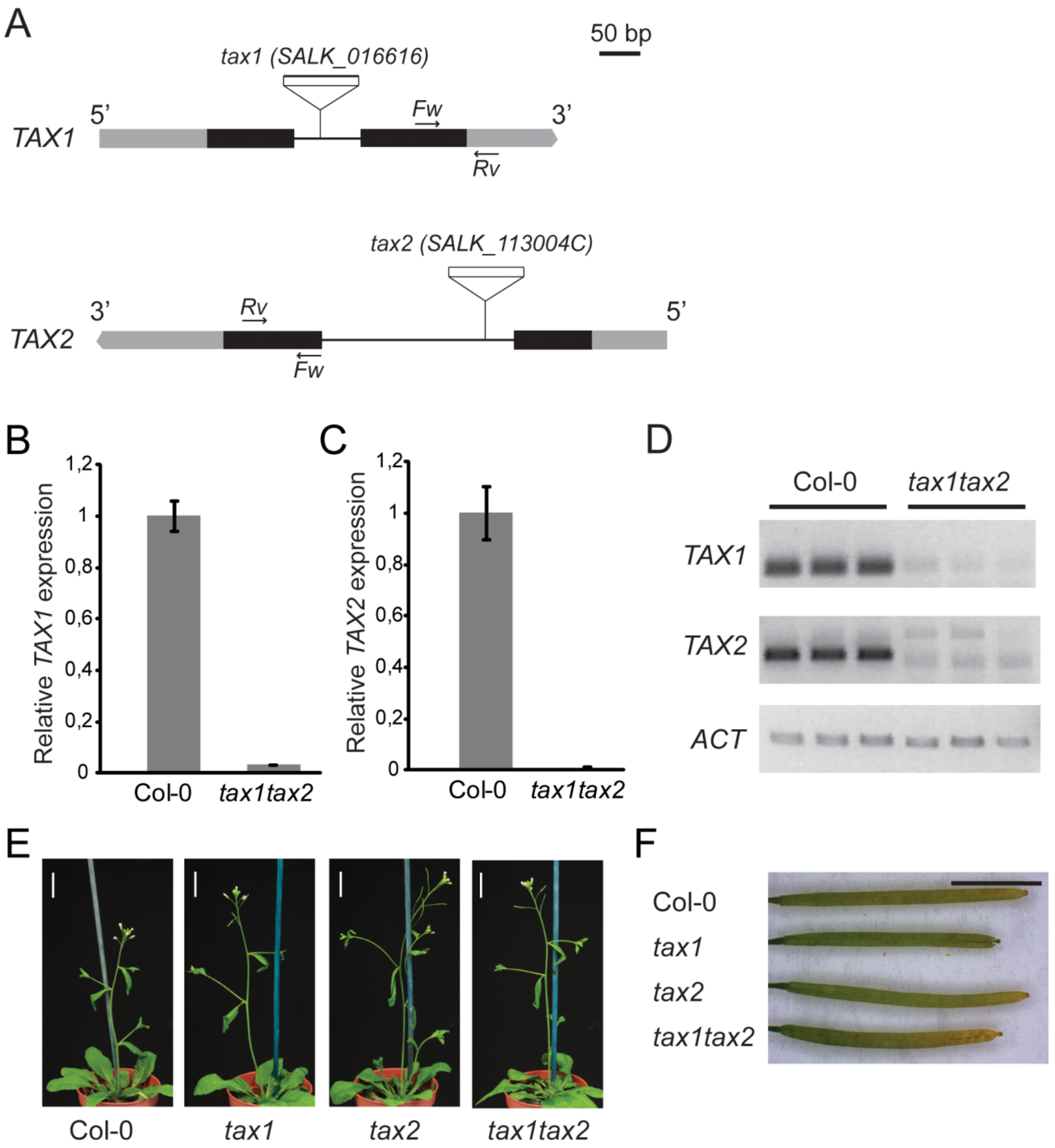

Supplementary Fig. S6. Generation and characterization of *tax* loss-of-function lines. (A) Location of primer pairs used for expression analysis. (B-C) qRT-PCR analysis of *TAX1* (A) and *TAX2* (B) expression in whole *tax1tax2* seedlings. *ACTIN* (At3g18780) was used as the reference gene. (D) RT-PCR analysis of *TAX1* and *TAX2* expression in *tax1tax2* paraclade junctions. (E) General morphology of adult single and double *tax1* and *tax2* mutants; scale bar, 2 cm. (F) Fruit morphology; scale bar is 1 mm.

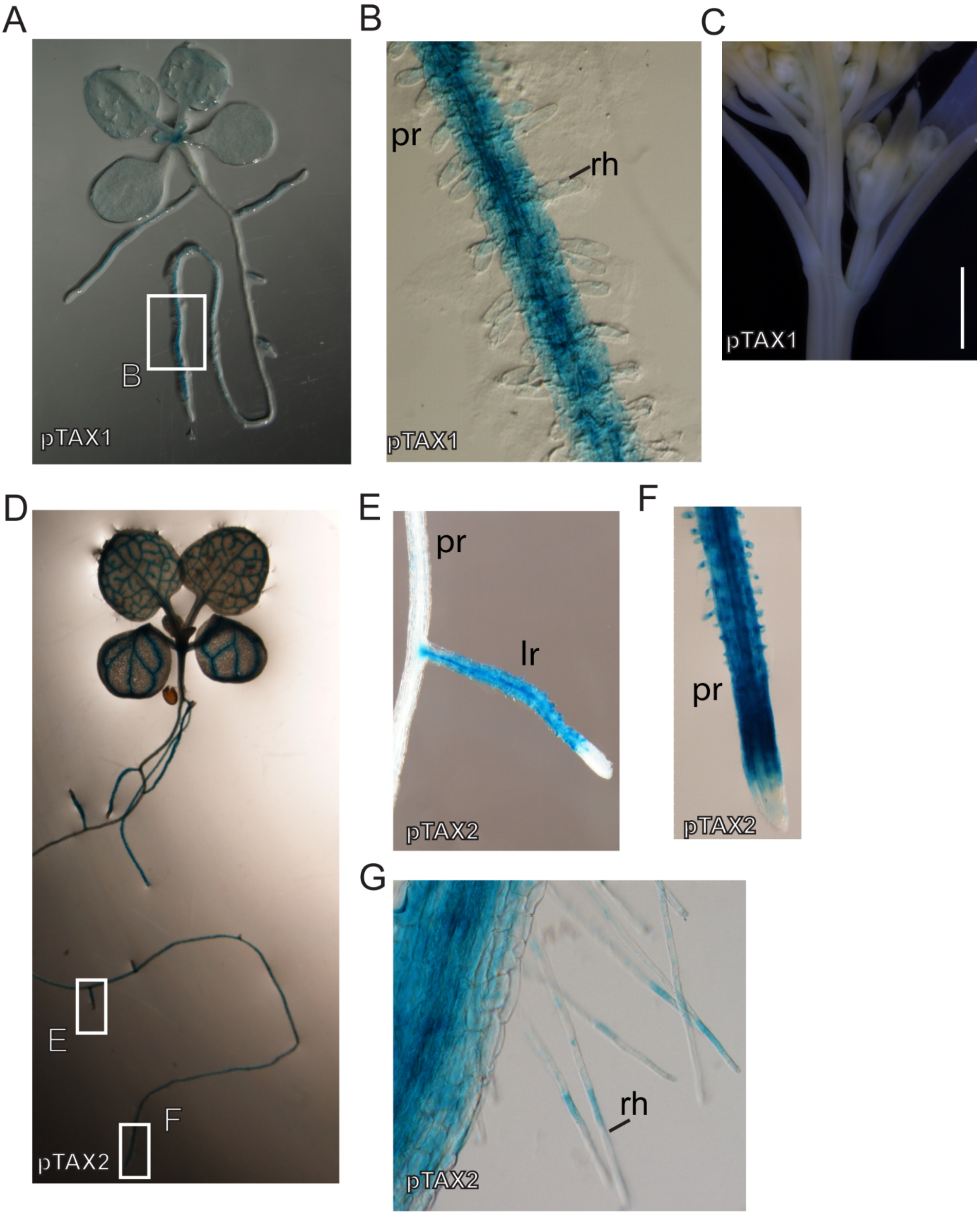

Supplementary Figure S7. *TAX1* and *TAX2* expression. Plants expressing a nuclear localized GUS-GFP fusion under control of the *TAX1* (A-C) and *TAX2* (D-G) promoter were used. (A-B) pTAX1-GUS activity in roots of 10-day-old seedlings, (C) pTAX1-GUS activity in the inflorescence of 28-day-old mature plants. (D-E) pTAX2-GUS activity in roots of 10-day-old seedlings. Abbreviations: pr, primary root; lr, lateral roots; rh, root hairs; c, cauline leaf. Scale bar, 1 mm.

Supplementary Table S1. Primers used in this study

ID		Sequence	Use
TAX1	Fw	GGGGACAAGTTTGTACAAAAAAGCAGGCTCCATGTGCGACGGAGATTGCCG	Cloning
TAX1	Rv	GGGGACCACTTTGTACAAGAAAGCTGGGTCTCMACAAGGGATCTTGGACATG	Cloning
TAX2	Fw	GGGGACAAGTTTGTACAAAAAAGCAGGCTCCATGGGAGATTGTAGACCTC	Cloning
TAX2	Rv	GGGGACCACTTTGTACAAGAAAGCTGGGTCTCMACAAGGAATGGAGTGGGTAAAC	Cloning
TAX1ΔSP	Fw	GGGGACAAGTTTGTACAAAAAAGCAGGCTCCATGATTATCTGGATCGTCGGATTG	Cloning
TbTAX-His	Fw	GGGGACAAGTTTGTACAAAAAAGCAGGCTTAATGGGGGAGTGCAGACCATTGGG	Cloning
TbTAX-His	Rv	GGGGACCACTTTGTACAAGAAAGCTGGGTATCAGTGGTGATGGTGATGATGGCAGGGTATTTGATGAATGG	Cloning
TAX1-Venus	Fw	GCTCCTCGCCCTTGCTCACCATACAAGGGATCTTGGACATG	Fusion PCR
TAX1-Venus	Rv	CATGTCCAAGATCCCTTGTATGGTGAGCAAGGGCGAGGAGC	Fusion PCR
TAX1-Venus	Rv	GGGGACCACTTTGTACAAGAAAGCTGGGTATTACTTGTACAGCTCGTCCATGCC	Fusion PCR
pTAX1	Fw	GGGGACAACTTTGTATAGAAAAGTTGTCTATGTTTTCTTGTGTCTCCAATGAGT	Cloning
pTAX1	Rv	GGGGACTGCTTTTTTGTACAAACTTGGGTGGCTCCGGCGGCGAGT	Cloning
pTAX2	Fw	GGGGACAACTTTGTATAGAAAAGTTGCCCCAAATTAAAAGGCCAAA	Cloning
pTAX2	Rv	GGGGACTGCTTTTTTGTACAAACTTGTTTGTGAAGACAAAAGACTAAG	Cloning
LBb1.3		ATTTTGCCGATTTCGGAAC	Genotyping
tax1	Fw	TGTCCAAGATCCCTTGTTGA	Genotyping
tax1	Rv	TCCAATACAAGTTATGTACATGGAAGA	Genotyping
tax2		CTCTTCCACAAGCCACAAGAG	Genotyping
tax2		AAAAACGACGGATTCATGATG	Genotyping
ACTIN	Fw	GTTGCACCACCTGAAAGGAAG	RT-PCR
ACTIN	Rv	CAATGGGACTAAAACGCAAAA	RT-PCR
TAX1	Fw	TGTTGTTATCATGCATATGT	qPCR
TAX1	Rv	CATGAACCACTCCATAACAT	qPCR
TAX2	Fw	ATCATTGGGACGGTATTGAGTTGTT	qPCR
TAX2	Rv	GGAGTGGGTAAACCAACGGAGG	qPCR
LOF1	Fw	CCCACAAAACTGGAACCTCA	qPCR
LOF1	Rv	CTCGGGTCCAATTGGTTAAA	qPCR
LOF2	Fw	TGTTACCAGTTCCTTGCTTCC	qPCR
LOF2	Rv	CATGCAATGTAATCGCCAAC	qPCR
CUC3	Fw	TCGAAAACGACCATTCACAC	qPCR
CUC3	Rv	AAGGTAGCTGATTTGGTTATGGA	qPCR
UBC	Fw	CTGCGACTCAGGGAATCTTCTAA	qPCR
UBC	Rv	TTGTGCCATTGAATTGAACCC	qPCR
PP2A	Fw	TAACGTGGCCAAAATGATGC	qPCR
PP2A	Rv	GTTCTCCACAACCGCTTGGT	qPCR
TCM: reverse primers were ambiguity coded to obtain both clones with stop codon (TGA) and without (GGA).			

Supplementary Table S2. Reporting metabolite data presented in this study.
See separate Excel file.

Supplementary Table S3. Primary metabolite profiling of Col-0 and *TAX1* overexpressing lines in leaf.
See separate Excel file.

Supplementary Table S4. Primary metabolite profiling of Col-0 and *TAX1* overexpressing lines in root.
See separate Excel file.
